# Serum albumin level as an indicator of response to Hepatitis B vaccination in dialysis patients: A systematic review and meta-analysis

**DOI:** 10.22088/cjim.8.4.250

**Published:** 2017

**Authors:** Mohammad Ebrahim Ghamar-Chehreh, Shahram Agah, Hossein Khedmat, Aghdas Aghaei, Seyed-Moayed Alavian

**Affiliations:** 1 Baqiyatallah Research Center for Gastroenterology and Liver Disease, Baqiyatallah University of Medical Sciences, Tehran.; 2 Colorectal Research Center, Iran University of Medical Sciences, Tehran, Iran.

**Keywords:** Hepatitis B vaccines, Seroconversion, Peritoneal dialysis, Renal dialysis

## Abstract

**Background:**

Hepatitis B (HB) vaccination is a recommended procedure in all dialysis patients, but its efficacy has not been perfect. In the current study, we aimed to conduct a comprehensive review of the literature to find and pool data of the randomized trials evaluating the impact of serum albumin levels on the immunogenicity of HB vaccination in dialysis patients.

**Methods:**

Literature searches were conducted by the Medline and Google Scholar. The key words used included ‘Hepatitis B’, ‘Vaccine’, ‘Dialysis’, ‘Hemodialysis’, and ‘Albumin’. Data of serum albumin levels regarding seroresponse to HB vaccine in clinical trials have been achieved and analyzed. Finally, data from 17 clinical trials have been pooled and analyzed.

**Results:**

One thousand six hundred eighty-two dialysis patients (1212 seroconverted) were included in the meta-analysis. Analysis of response to HB vaccination in our dialysis population showed a significant relationship to their serum albumin levels (p<0.001, z= 5.23). Regarding the dialysis mode, serum albumin level was only a significant interfering factor in hemodialysis patients versus continuous ambulatory peritoneal dialysis (CAPD) (HD group: p<0.001, I^2^=88.5%, χ^2^=95.28 (d.f. = 11); CAPD (±HD) group: χ^2^= 2.21; P=0.697, I^2^= 0%, d.f.= 4).

**Conclusion:**

The data showed a significant effect for the levels of serum albumin on the immunogenicity of HB vaccine in dialysis patients. Moreover, stratification of data upon dialysis mode showed that this association is only available for hemodialysis patients, and not those on peritoneal dialysis.

**C**ontrolling viral infections in dialysis units is a major conflict in the management of end-stage renal disease (ESRD) patients which can significantly compromise success of the procedure and negatively affects survival of the patients (1). There are hygienic precautions which have been developed and published by different societies, and reports are indicative of their feasibility in the prevention of blood-borne infections especially through nosocomial transmission (2). Nevertheless, despite the significant improvement in the burden of viral hepatitis B infection in dialysis patients, it persists as a major health problem especially in developing countries and in the region with high endemicity for HBV infection. Hepatitis B virus (HBV) infection is a frequent infection in dialysis patients and vaccination has been recommended as an indispensable part of preventive strategies for protecting dialysis patients against HBV infection (3). However, it has been demonstrated that despite adherence to all the precautions as well as vaccination, there are still a considerable number of dialysis patients susceptible to infection with HBV.

This shows that the effectiveness of HBV vaccination in dialysis patients is associated with faults, and since this happens in only a proportion of patients, finding factors significantly influencing immunogenicity of HBV vaccination in this population could be considered of utmost importance (4).

Several conducted studies with strong methodologies have been performed to find potential factors affecting response to HBV vaccines in dialysis patients, and different studies have found a wide range of them, although several controversies have also been reported. To pool the existing data from different clinical trials to have a comprehensive analysis of the existing data, several meta-analyses have been done through which several interesting findings have been revealed. 

In the field of immunogenicity of HBV vaccines in dialysis patients, Fabriziet al. (3) have conducted the most perfect meta-analyses in whose studies potential impacts of a broad range of factors on response to HBV vaccination in dialysis patients have been investigated: Erythropoietin use, diabetes mellitus, dialysis mode, mode of vaccine administration (intradermal versus intramuscular), levamisole use, use of granulocyte macrophage-colony stimulating factor on immunological, thymopentin use, and other adjuvant use, the effect of age and nutritional status of dialysis patients on the immunogenicity of HBV vaccine were among them. 

Although nutritional status has been investigated in a meta-analysis by Fabrizi et al. [reff], serum albumin was only added only in 5 of the included studies. In the current meta-analysis, using update data from the literature, we aimed to separately evaluate the effects of serum albumin levels on the response to HBV vaccination in dialysis patients.

## Methods

Search strategy and data acquisition: Literature searches were performed through the National Library of Medicine’s (Medline) database, and Google Scholar; the latter has been especially used to find relevant citations of the randomized controlled trials of interest; as well as search of specific journals were performed to identify all the associated evidence. The key words used included ‘hepatitis B’, ‘vaccine’, ‘dialysis’, ‘hemodialysis’, ‘haemodialysis’, and ‘albumin’. The search has also been repeated using the reference lists of the related reviews and meta-analyses. Among the relevant articles, all the found clinical trials representing comparative analysis of the ‘serum albumin’ for the response to hepatitis B vaccination were in English. There was no restriction in the time of publication for our search, and all the studies fulfilling the inclusion criteria were included into the analysis, irrespective of their publication year.

**Inclusion and exclusion criteria:** To get included in this systematic review, studies should have fulfilled the following criteria: (1) they had to be available as full text (In cases the full text was not available, we contacted the corresponding author with requests to send us the full text papers; (2) they should have been conducted using prospective approach; (3) their data are presented in a form that could be used to construct a database for meta-analysis were considered eligible for inclusion. The vaccines employed in the trials could be either plasma-derived or recombinant DNA preparations. The administered dosages or follow-up times or vaccination routes were not subjected to any preferable inclusion or exclusion. Studies were excluded if: (1) they reported no data on serum albumin levels separately for dialysis patients responding versus non-responding to HB vaccines; (2) a study has not reported data of serum albumin in a mean±SD mode, e.g. presenting median of serum albumin; (3) trials were published as abstracts. 

**End point:** The association of mean±SD serum albumin levels has been associated with seroresponse to HB vaccine in clinical trials. When both seroprotection and seroconversion had been reported by the included trials, seroconversion was used as the end-point. 

**Literature review:** Our search identified 22 full text studies that have been achieved and reviewed. After excluding studies not fulfilling the inclusion criteria, 17 clinical trials (5-21) remained representing the 1682 dialysis/chronic kidney disease patients that were included in our meta-analysis (figure 1).

**Statistical methods: **The meta-analysis was performed using a random effects approach. The effect size is the main outcome of each trial which was standardized to one SD unit calculating a standardized mean difference (SMD) by meta-analysis for each dialysis mode and total analysis. Test of heterogeneity between the studies was assessed using the I^2^ statistics, which described the proportion of total variation across studies that was the result of heterogeneity rather than chance. Statistical heterogeneity was present, defined as P≤0.05 or I^2^> 50%. All statistical pooling was conducted using “metan” user-written commands. The meta-analysis has been performed using software Stata Version 9 (Statacorp, TX, USA). 

## Results

**Patient characteristics:** Table 1 summarizes the demographic and clinical characteristics of the studies and their subjects enrolled in the current meta-analysis. All of the included clinical trials were published in English and the year of publication ranged from 1995 to 2012. Seven out of the seventeen studies (41%) were from the Middle East (4 from Turkey, and one each from Iran, Egypt and Saudi Arabia) and the remaining studies were from Canada (3), China and Taiwan (3) and Spain & UK (1 each). In 11 (64.7%) studies, all patients were under hemodialysis while in only two (11.8%) patients under CAPD were investigated, in 3 (17.6%) studies, both of the dialysis modes were used, and in the remaining one study, subjects with chronic kidney disease (CKD). The mean age of the participants in the included cohorts ranged from 44 to 60 years, the mean duration of dialysis also ranged from 3.4 to over 80 months while the gender distribution ranged from 35% to 75% males. In two of the studies, the intradermal mode of vaccination has been used besides the intramuscular (IM) mode which was not possible to compare their results separately (table 2). All of the studies have used recombinant vaccines, and only in one study, a share of patients received plasma-derived vaccine. In all but one (20 µg) of the IM vaccine administrations, 40 µg doses of vaccine have been administered; although in two studies, a dose of 80 µg vaccine and in another one a 20 µg dose had also been used, in a share of patients. In intradermal administration, the dose was 5 µg vaccine dose of vaccine in one study and 20 µg in another. Schedule of vaccination in four of the studies was 3 times (with different time intervals) while the rest 4-times (0, 1, 2, 6).

**Table 1:** Demography of the included clinical trial participants

**First author**	**Participant number**	**Dialysis mode**	**Age (mean±SD** * **)**	**Gender male (%)**	**Duration of dialysis (months)**
Charest, canda, 2000 (8)	97	HD	52±2 (ID*) 46±2 (IM*)	73(75)	3.4±1.0 (ID) 4.8±2.0 (IM)
Waite, canda, 1995 (10)	77	HD	NA (for total)	49(64)	NA (for total)
DaRoza, canada, 2003 (16)	165	CKD	60±15	106(46)	NA
Chow, China, 2010 (5)	87	CAPD	60±11	51(59)	5.8 (median)
Kovacic V., Croatia, 2002 (20)	30	HD	60±9	20(67)	4.2±3.6 years
Ibrahim, Egypt, 2006 (13)	29	HD	46±11	19(66)	80±59
Eleftheriadis, Greece, 2010 (21)	66	HD	61±13	44(67)	NA
Roozbeh, Iran, 2005 (18)	62	HD	NA(for total)	37(60)	NA
Al Saran, Saudi Arabia, 2014 (19)	144	HD	51±15	78 (54)	3.3 years
Fernandez, Spain, 1996 (12)	64	HD	58 (average)	48(75)	42±4
Liu, Taiwan, 2005 (9)	69	HD& CAPD	52±16 (CAPD) 61±11 (HD)	28(41)	43±33 (CAPD) 60±49 (HD)
Lin, Taiwan, 2012 (14)	156	HD& CAPD	NA(for total)	64(41)	NA
Kara, Turkey, 2004 (6)	34	HD	44±15	19(56)	27±15
Afsar, Turkey, 2009 (7)	188	HD	NA* (for total)	66(35)	NA (for total)
Dervisoglu, Turkey, 2011 (11)	33	CAPD	49±12	20(61)	28±23
Sit. Turkey, 2007 (15)	64	HD	NA (for total)	31 (48)	NA (for total)
Bel’eed, UK, 2002 (17)	317	HD& CAPD	NA(for total)	152(70)	NA

* Ref: reference number; CAPD; continuous ambulatory peritoneal dialysis; HD: hemodialysis; ID: intra-dermal; IM: intramuscular;

** SD: standard deviation; CAPD; continuous ambulatory peritoneal dialysis; HD: hemodialysis; NA: not available; ID: intra-dermal; IM: intramuscular;

**Table 2:** Vaccination details in the included clinical trials

**Author**	**Vaccination mode**	**Vaccine type**	**Vaccine dose**	**Schedule (months)**
Charest, canada, (8)	ID*&IM	Recombinant (Engerix-B)	40mcg (IM); 5 mcg (ID)	0,1,2,6
Waite, canada (10)	IM	Recombinant (Engerix-B)	40 mcg	0,1,2,6
DaRoza, canada (16)	IM	Recombinant & plasma derived	20&40&80 mcg	0,1,6
Chow, China, (5)	IM	Recombinant (Engerix-B)	40mcg & 80mcg	0,1,6
Kovacic V. Croatia (20)	IM	Recombinant (Engerix-B)	40 mcg	
Ibrahim, Egypt (13)	IM	Recombinant (Engerix-B)	40 mcg	0,1,2,6
Eleftheriadis, Greece (21)	IM	Recombinant (Engerix-B)	40 mcg	
Roozbeh, Iran (18)	IM&ID	Recombinant (Herberbiovac-HB)	40mcg(IM); 20 mcg (ID)	0,1,4
Al Saran, Saudi Arabia (19)	IM	Recombinant (Engerix-B)	40 mcg	
Fernandez, Spain (12)	IM	Recombinant (Engerix-B)	20*2(40) mcg	0,1,6
Liu, Taiwan (9)	IM	Recombinant (Engerix-B)	40 mcg	0,1,2,6
Lin, Taiwan (14)	IM	Recombinant (Engerix-B)	40 mcg	0,1,2,6
Kara, Turkey (6)	IM	Recombinant (Engerix-B)	40mcg	0,1,2,6
Afsar, Turkey (7)	IM	Recombinant	-	0,1,2,6
Dervisoglu, Turkey (11)	IM	Recombinant (Euvax B)	20 mcg	0,1,2,6
Sit, Turkey (15)	IM	Recombinant (Hepavax)	40 mcg	0,1,2,6
Bel’eed, United Kingdom (17)	IM	Recombinant (Engerix-B)	40 mcg	

mcg: micrograms; ID: intra-dermal; IM: intramuscular;

**Summary of outcome:** Analysis of response to HB vaccination in our dialysis population showed a significant relation to their serum albumin levels (p<0.001, z=5.23; figure I). As well, a significant heterogeneity has been detected in the analysis of the included studies (p=0.048; heterogeneity χ^2^=6.06 (d.f.=2) I^2^ (variation in SMD attributable to heterogeneity)= 88.5%).

**Reanalysis regarding dialysis mode:** Then, we conducted a reanalysis of the pooled data from our studies regarding the dialysis mode (hemodialysis versus CAPD (±HD) to evaluate potential differential impact of serum albumin level on HB vaccine response. Serum albumin level was the only significant interfering factor in hemodialysis patients (figure II; HD group: p<0.001, I^2^=88.5%, χ^2^=95.28 (d.f.=11); CAPD (±HD) group: χ^2^= 2.21; P=0.697, I^2^= 0%, d.f.= 4)

**Figure I:**
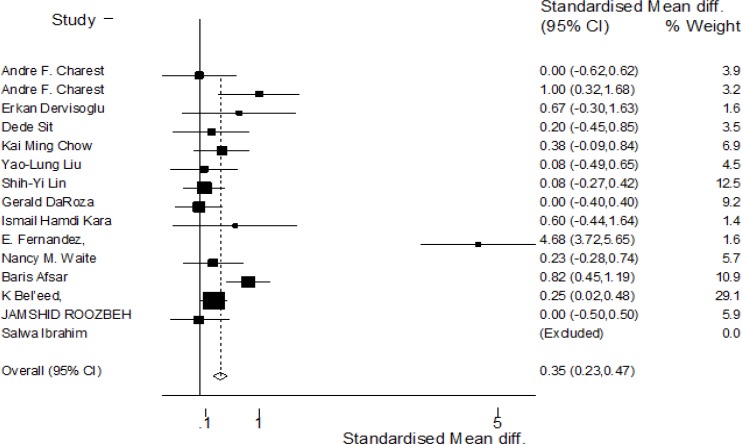
Forest plot: Meta-analysis of the association between serum albumin levels and seroresponse to hepatitis B vaccine

**Figure II:**
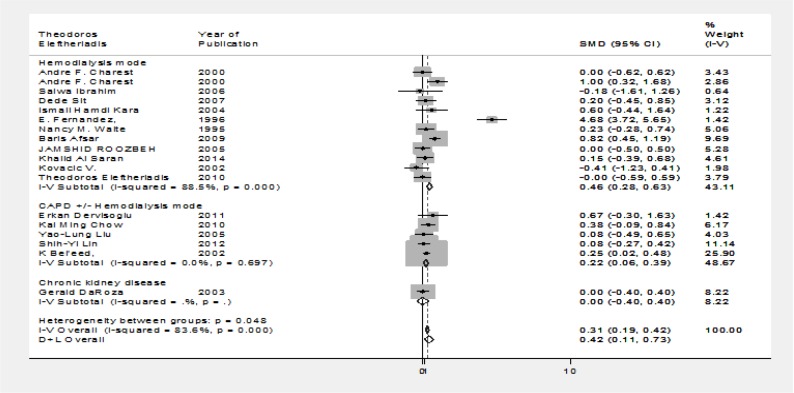
Forrest plot: reanalysis of the data separating data based on the therapy modality

## Discussion

HBV vaccination has been an essential part of immunization in chronic kidney disease patients especially those under maintenance dialysis, and several experts and organizations have recommended its administration in this patient population (3, 22, 23). The compromised immune system as he well as the therapeutic procedures used for these patients (e.g. repetitive injections, blood transfusions and cross-contamination through environmental and procedural devices) puts these patients in a very vulnerable condition. On the other hand, immune response to vaccination in these patients is also impaired (4) and despite the comprehensive endeavors which have been made to enhance the efficacy of immunization in this population, still a considerable number of patients do not well respond to vaccination (23, 24). For that same reason, in a previously published paper, we recommended that immunization in dialysis patients more likely to be performed through on individualized approach (4), and firstly for this purpose, we need to significantly recognize the factors that interfere with response to HBV vaccination. 

There is a large amount of evidence in the existing literature coming from the randomized controlled trials or meta-analyses that propose the significant effects of some factors on the response rates to HB vaccination in kidney disease patients. Maybe the most comprehensive systematic studies on this issue were published by Fabrizi et al. which investigated quite a number of potential interfering factors in this era, providing invaluable data for potential future endeavors directing at individualization of vaccination for dialysis population. Some of the findings of the meta-analyses by Fabrizi et al. on the effects of different factors upon immunogenicity of HB vaccination in dialysis patients include: no significant effects while taking erythropoietin (Epo) (25), similar findings were observed for some other adjuvants (26); while significant effects for uring treatment with levamisole (27), granulocyte macrophage-colony stimulating factor (28) and thymopentin use (29). Moreover, seroresponse of patients under hemodialysis versus those on maintenance peritoneal dialysis had been compared with no significant difference detected (30); but intradermal administration of HB vaccine was associated with a significantly higher vaccine response in dialysis patients (31). Diabetes mellitus (32) and older age (33) were also significantly associated with poorer response to HB vaccination.

Among the meta-analyses performed by Fabrizi et al., maybe the most relevant one to our current research is a recent meta-analysis on the potential effect of nutritional status of dialysis patients which showed a significant impairing effect on immune response to HB vaccine (34). In this meta-analysis, data from studies comparing a range of indicators of nutritional status with high focus on serum albumin levels have been pooled and analyzed and finally the authors have reported a significant impact of poor nutritional status on poor seroconversion after HB vaccination (34). However, the number of the included studies in that meta-analysis (due to using categorized levels of serum albumin levels for comparisons) was quite more limited than ours, and also not all of the studies had investigated serum albumin levels as the indicator of nutritional status. But in the current study, serum albumin levels were the only factors investigated, and this will make our study more particular. Moreover, in this study while is we used the mean±SD of serum albumin levels in patients who did or did not experience seroconversion. This resulted in a larger number of clinical trials fulfilling the inclusion criteria for the meta-analysis.

The main finding of this study which is the impaired impact of low serum albumin levels on the immunogenicity of HB vaccine is of utmost important. This observation recommends that physicians who want to immunize their dialysis patients should have a good idea on their patients’ nutritional status and serum albumin levels; and if their condition is not satisfactory, they should be treated so their nutritional status improve before any attempt to vaccination. 

Another very interesting finding of the current study is that we surprisingly found that mean±SD serum albumin level is only significant in patients on hemodialysis and neither those on peritoneal dialysis nor in chronic kidney disease patients without dialysis therapy. One explanation for this observation could be because of the direct blood purification during a hemodialysis session, the antigens of the HB vaccine that should elicit an immune response would also be washed out in the absence of albumin molecules which might attach to them thus preventing them from simply getting cleared from the blood. This finding is of practical significance as well which proposes no surveillance of peritoneal dialysis patients for serum albumin levels before HB vaccination.

Despite some limitations, we believe that the results of the current study add significant data to the literature. This study provides the strongest evidence on the significance of serum albumin levels on the immunogenicity of HB vaccination in kidney disease patients. In conclusion, this meta-analysis showed a significant effect on the levels of serum albumin in the immunogenicity of HB vaccine of dialysis patients. Furthermore, stratification of data upon dialysis mode showed that this association is only available for hemodialysis patients, and not to those on peritoneal dialysis. Our data help physicians to a more particularly individualized immunization of their dialysis population. Future studies directing to find other interfering factors are recommended.

## Funding:

 This meta-analysis was not supported by any pharmaceutical company. The source of support in this study is a grant by Baqiyatallah University of Medical Sciences, Tehran, Iran.
